# The role of the assessment policy in the relation between learning and performance

**DOI:** 10.1111/medu.13487

**Published:** 2017-12-11

**Authors:** Rob Kickert, Karen M Stegers‐Jager, Marieke Meeuwisse, Peter Prinzie, Lidia R Arends

**Affiliations:** ^1^ Department of Psychology, Education and Child Studies Erasmus School of Social and Behavioural Sciences Erasmus University Rotterdam Rotterdam the Netherlands; ^2^ Institute of Medical Education Research Rotterdam Erasmus MC University Medical Center Rotterdam Rotterdam the Netherlands; ^3^ Department of Biostatistics Erasmus MC University Medical Center Rotterdam Rotterdam the Netherlands

## Abstract

**Context:**

Optimising student learning and academic performance is a continuous challenge for medical schools. The assessment policy may influence both learning and performance. Previously, the joint contribution of self‐regulated learning (SRL) and participation in scheduled learning activities towards academic performance has been reported. However, little is known about the relationships between SRL, participation and academic performance under different assessment policies.

**Objectives:**

The goal of this study was to investigate differences in average scores of SRL, participation and academic performance of students under two assessment policies: (i) a conjunctive lower stakes, lower performance standard (old) assessment policy and (ii) a compensatory higher stakes, higher performance standard (new) assessment policy. In addition, this research investigated whether the relationships between academic performance, SRL and participation are similar across both assessment policies.

**Methods:**

Year‐1 medical students (i) under the old assessment policy (*n* = 648) and (ii) under the new assessment policy (*n* = 529) completed the Motivated Strategies for Learning Questionnaire on SRL, and additional items on participation. Year‐1 performance was operationalised as students’ average Year‐1 course examination grades. manova and structural equation modelling were used for analyses.

**Results:**

Generally, students under the new assessment policy showed significantly higher Year‐1 performance, SRL and participation, compared with students under the old assessment policy. The relationships between Year‐1 performance, SRL and participation were similar across assessment policies.

**Conclusions:**

This study indicates that the higher academic performance under a compensatory higher stakes, higher performance standard assessment policy, results from higher SRL and participation, but not from altered relationships between SRL, participation and performance. In sum, assessment policies have the potential to optimise student learning and performance.

## Abstract 2

This study indicates that student learning and performance are sensitive to changes in characteristics of the assessment policy, such as higher stakes and higher performance standards.

## Introduction

Optimising student learning and academic performance is a continuous challenge for medical schools. Because several studies have shown that ‘assessment drives learning’,^1^, ^2^, ^3^ modifying the assessment policy may be an efficacious way to improve student learning and to enhance academic performance (e.g. average grades). For instance, there is empirical evidence that performance is superior on tests with higher stakes^4^, ^5^, ^6^ or higher performance standards.^7^, ^8^ Another line of research has shown that self‐regulated learning (SRL^9^, ^10^) and participation in scheduled learning activities^11^, ^12^ are key predictors of academic performance, and reported on their joint contribution.^13^ However, it is not known how assessment policies affect SRL, participation and performance for medical students. This study filled this gap by investigating whether average SRL, participation in scheduled learning activities and academic performance differ under two assessment policies, which vary in terms of stakes and performance standards. There is also a lack of research on how SRL and participation relate to academic performance under different assessment policies. As a starting point, we used a tested and cross‐validated integrated model of SRL, participation and Year‐1 medical student performance that was developed under a conjunctive, lower stakes, lower performance standard assessment policy.^13^ We tested whether this model could be cross‐validated in a new sample of students who were subjected to a compensatory, higher stakes, higher performance standard assessment policy.

### Self‐regulated learning, participation and academic performance

Self‐regulated learners are able to (i) control their own effort and motivation, (ii) reflect on their learning process and adapt this process when necessary, and (iii) use proper behavioural strategies for learning, for instance summarising the literature.^10^

There is strong empirical evidence for the association of SRL with academic performance.^4^, ^10^, ^13^, ^14^, ^15^, ^16^, ^17^, ^18^ For instance, higher levels of several motivational constructs, such as intrinsic goals, self‐efficacy and task value, have been shown to be associated with improved academic performance.^10^, ^13^, ^16^, ^17^, ^18^ The same holds for learning strategies such as metacognitive self‐regulation, elaboration, organisation, time management and effort regulation.^13^, ^16^, ^17^, ^18^ Composite scores of SRL are positively associated with academic performance as well.^4^, ^14^, ^15^

In addition to SRL, participation in scheduled learning activities is another important predictor of academic performance.^11^, ^12^, ^19^ Students’ physical presence at lectures or other modes of instruction is a crucial predictor of higher academic performance.^11^ More individual study time also predicts higher academic performance.^12^, ^19^

A study by Stegers‐Jager, Cohen‐Schotanus and Themmen^13^ showed the joint contribution of SRL and participation towards academic performance. Self‐regulated learning (SRL) was operationalised as ‘motivational beliefs’ and ‘learning strategies’ and measured with the Motivated Strategies for Learning Questionnaire, which we also used in the current study.^13^ ‘Motivational beliefs’ consisted of ‘value’ and ‘self‐efficacy’, whereas ‘deep learning strategies’ and ‘resource management’ were indicators of ‘learning strategies’. Positive associations between these components of SRL, participation in scheduled learning activities and academic performance were found, which indicated that higher SRL is related to higher participation and higher academic performance.^13^ In addition, deep learning strategies showed a weaker but statistically significant negative direct link to average grade.^13^ In other words, although deep learning is positively associated with academic performance through resource management and participation, when controlling for this positive pathway, there is a negative association between deep learning and academic performance. In sum, previous research has shown that it is valuable to consider the joint contribution of SRL and participation towards academic performance.

### The role of assessment policies

Several studies have shown that raising the stakes (i.e. higher consequences of performance) is associated with superior academic performance^4^, ^5^, ^6^ and increased motivation.^4^, ^6^, ^20^, ^21^ Higher performance standards (i.e. higher demands in order to pass) have also been associated with increased academic performance.^7^, ^8^ The available research on the interrelationships between SRL and academic performance shows that when the stakes are raised, motivation becomes less predictive of performance in both high‐school^20^ and college students.^4^, ^22^ By contrast, metacognition, as well as overall measures of learning strategies, are more important predictors of performance when the stakes are higher.^4^, ^22^

However, none of these investigations focused on medical students or included participation. In addition, studies investigating the effects of higher stakes,^4^, ^5^, ^6^, ^20^, ^21^, ^22^ compared tests with no consequences (e.g. test does not count as part of the grade) to tests with consequences (e.g. test counts as part of the grade). In this study, we compared tests with consequences (e.g. students need to obtain all Year‐1 credits within 2 years) to tests with even higher consequences (e.g. students need to obtain all Year‐1 credits within 1 year).

### The current research

In this study, we investigated the effect of assessment policies on SRL, participation in learning activities and academic performance of Year‐1 medical students. Firstly, we compared the average scores on SRL, participation in learning activities and academic performance of student cohorts in the two assessment policies. We hypothesised that motivational beliefs and academic performance would be superior under higher stakes and higher performance standards. Based on the available literature, we were not able to formulate any hypotheses on learning strategies and participation.

Secondly, we examined whether the relationships between SRL, participation in learning activities and academic performance were similar under different assessment policies. Therefore, we tested whether the model that was developed by Stegers‐Jager et al.^13^ was invariant for students under both assessment policies. In the case of higher stakes and higher performance standards, we expected that motivational beliefs would show weaker relationships with academic performance and that learning strategies would show stronger relationships with academic performance, compared with the lower stakes and lower performance standard assessment policy.

## Method

### Context

Both the initial study by Stegers‐Jager et al.^13^ and the current study were performed with Year‐1 students at the Erasmus MC Medical School, Rotterdam (the Netherlands). The curriculum consists of a 3‐year Bachelor's programme, followed by a 3‐year Master's degree course. Year 1 of the Bachelor programme consists of three thematic blocks and nine written exams. These exams are graded on a 10‐point scale (1 = poor to 10 = perfect) and consist of both closed and open‐ended questions. There are four types of learning activities: (i) large‐group learning (lectures and patient demonstrations; 8 hours a week), (ii) small‐group learning (skills training and tutorials; 8 hours a week), (iii) guided individual study (study assignments; 16 hours a week) and (iv) unguided individual study (8 hours a week). The small‐group learning is compulsory for approximately a quarter of the meetings; the other learning activities are voluntary.

The only major curriculum alteration over the past years was the change in the assessment policy in 2014. The courses and the content of the curriculum have remained stable. The change to the assessment policy was made with the intention to accelerate academic progress of Year‐1 students.^23^ In the previous lower stakes, lower performance standard, conjunctive (old) assessment policy,^24^ students needed to obtain a sufficient grade (i.e. at least 5.5 out of 10) on each of nine examinations. Students were required to obtain 40 out of 60 possible Year‐1 credits within the first year of enrolment in order to be allowed to proceed to the second year. After 2 years, all 60 Year‐1 credits needed to be obtained to prevent academic dismissal. Students thus had three resit opportunities per examination, one in the first year and two more in the second year. In the new, higher stakes, higher performance standard, compensatory (new) assessment policy,^23^, ^25^ obtainment of all 60 Year‐1 credits within the first year of enrolment is compulsory in order for students to prevent academic dismissal. Therefore, per examination there is only one resit opportunity, resulting in higher stakes per individual examination, because the consequences of failing an assessment have risen. Also, an average grade of at least 6.0 is required; two grades of 5.0–5.49 are allowed under the condition that they are not obtained in the same thematic block. Thus, compensation is allowed, albeit minimal.

Hence, there are differences between the two assessment policies both in terms of the consequences of not obtaining all credits within the first year (i.e. the stakes) and in terms of the required grades in order to pass (i.e. the performance standards). It should be noted that another way for students to prevent academic dismissal once, is to drop out before February, in which case students are allowed to re‐enter Year 1 of the Bachelor programme the next year. All the assessments are developed by an expert team in order to assure the quality and consistency of the assessments. Additionally, the Hofstee's method of standard setting is used for determining the pass or fail score per assessment (see Bandaranayke 2008^26^ for a detailed description of the Hofstee method). The Hofstee method has been applied similarly under both assessment policies. Consequently, both the content of the assessments and the average pass or fail scores have remained stable over the years. To balance out possible fluctuations in assessment characteristics that may have remained despite these precautions, we used two cohorts for the old assessment policy, as well as two cohorts for the new policy.

### Participants and procedure

The participants in this study were Year‐1 medical students, who enrolled in September 2008 and 2009 (old cohorts) or 2014 and 2015 (new cohorts). Each year, 2 months after enrolment, all Year‐1 students were invited to voluntarily complete an online survey on SRL and participation in learning activities, which took 15–20 minutes. The students automatically received feedback on the basis of their SRL scores, providing information about their strengths and weaknesses, as well as recommendations for improvement. Students were informed about the study, in which they could voluntarily participate with guaranteed confidentiality. Because there was no plausible harm to participants in this study, the ethical committee of the Department of Psychology, Education and Child Studies of Erasmus University Rotterdam deemed further approval of a Medical Ethical Evaluation committee to be not required. Prior to analyses, all data were coded and saved without directly identifiable information.

### Measures

**Figure 1:**
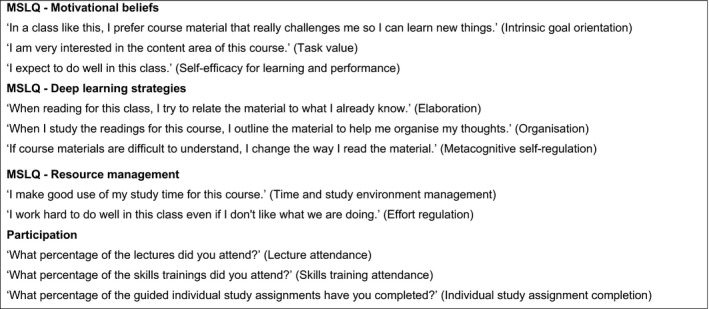
Example items from selected subscales of the Motivated Strategies for Learning Questionnaire, and participation items

Self‐regulated learning (SRL) was measured with the Motivated Strategies for Learning Questionnaire (MSLQ^27^), a thoroughly tested tool^28^ that is reliable and useful in predicting academic performance,^17^, ^18^ and proven appropriate in the medical context.^29^ In line with Stegers‐Jager et al.,^13^ we used a Dutch translation of the MSLQ^30^ for measuring motivational beliefs (subscales of intrinsic goal orientation, task value and academic self‐efficacy), deep learning strategies (subscales of elaboration, organisation and metacognitive self‐regulation) and resource management (subscales of time and study environment, and effort regulation) (see Fig. 1 for example items). Items are scored on a 7‐point Likert scale (1 = *not at all true of me*; 7 = *very true of me*). Some items were minimally adapted to make them more suited for the specific medical school context, for instance by changing the word ‘course’ to ‘theme’ (c.f. Stegers‐Jager et al.^13^).

Students were also asked to report on their participation in scheduled learning activities using three 5‐point scale items about percentages of lecture attendance, skills training attendance and individual study assignment completion (see Fig. 1).

### Outcome measure: Year‐1 performance

At the end of the academic year, we obtained students’ grades for their first attempt at all 9 Year‐1 course examinations from the university student administration system. Next, we calculated Year‐1 performance as the unweighted average of the grades for all students who earned at least seven grades, regardless of whether these grades were sufficient or not.

### Statistical analyses

After we screened for accuracy of data entry and missing values, and checked the study variables for normality, we calculated descriptive statistics and Pearson correlations, and Cronbach alphas for the subscales of the MSLQ. To examine differences in SRL, participation and performance between the assessment policies a manova 31 was performed. Analyses were performed using IBM spss Statistics for Windows Version 23.0 (IBM Corp., Armonk, NY, USA).^32^ We checked Box's *M* to assess whether necessary assumptions were met. Next, we calculated Pillai's Trace for the overall model. In case of a significant outcome of the multivariate test, we performed univariate anovas on the separate dependent variables. We calculated *F‐*values and Cohen's *d* (0.20 = small effect size; 0.50 = medium effect size; 0.80 = large effect size^33^) for the individual dependent variables.

We performed a multi‐group analysis with structural equation modelling (SEM),^34^ using amos 22.0 (SPSS, Inc., Chicago, IL, USA),^35^ with students under the old assessment policy as the first group and students under the new policy as the second group. Structural equation modelling (SEM) combines factor analysis with regression, by creating latent constructs from observed scale scores, and then regressing these latent constructs on each other.^34^ The goal of the multi‐group SEM was to investigate whether there is structural invariance, meaning that the structural regression paths between the latent constructs are similar in both groups^34^ (e.g. whether the regression path between the latent constructs deep learning and resource management is similar between groups). A necessary condition in order to assess structural invariance is measurement invariance. Measurement invariance means that the factor loadings (i.e. the connections between the latent constructs and their corresponding observed scale scores) are similar between groups.^36^ In other words, measurement invariance indicates whether the same construct is being measured across the specified groups (e.g. whether the observed scale scores for time management and effort regulation have similar loadings on the latent construct resource management in both groups).

In order to assess whether the factor loadings and structural paths were identical across groups, we added constraints in a stepwise manner. Firstly, to test measurement invariance, we constrained all factor loadings, error covariances and covariances to be equal across groups. Secondly, to test structural invariance, we constrained the structural paths to be equal, in addition to the constraints of the first step. Maximum likelihood estimation was used to estimate model parameters and a chi‐squared test to assess model fit was supplemented by the comparative fit index (CFI), the standardised root mean squared residual (SRMR), the root mean square error of approximation (RMSEA) and the Akaike information criterion (AIC). Because the chi‐squared test is strongly affected by sample size, the additional measures are necessary for evaluating model fit.^34^ In general, the following results for these fit indices are considered good: a CFI ≥ 0.95, an SRMR ≤ 0.08 and an RMSEA ≤ 0.06.^37^

## Results

### Respondents

The inclusion criteria for the study were that students completed the questionnaire, and attended at least seven out of nine possible assessments. For the 2008 and 2009 cohorts, 82% out of 817 Year‐1 students completed the questionnaire, and 93% of the 817 students obtained at least 7 grades. In total 79% of the students met both inclusion criteria (*n* = 648, 35% male, *M*_AGE_ = 19.3 years, SD_AGE_ = 1.56 years). For the 2014 and 2015 cohorts, 79% out of 822 students completed the questionnaire, and 81% of the students obtained at least 7 grades as well. In total, 64% of the students met both inclusion criteria (*n* = 529, 33% male, *M*_AGE_ = 19.0 years, SD_AGE_ = 1.82 years). All respondents answered all items of the questionnaire.

### Descriptive statistics

The descriptive statistics for the study variables are presented in Table 1 and the correlations between the study variables are presented in Table 2. The Cronbach's alphas of the subscales for all four cohorts combined ranged from 0.61 to 0.87 (see Table 2). Overall, the correlations between the study variables were slightly lower under the new assessment policy, compared with the old policy.

**Table 1:** Descriptives, p‐values and effect sizes for the study variables (old cohorts [*n* = 648] and new cohorts [*n* = 529])

	Variable	*M*_old_	SD_old_	*M*_new_	SD_new_	p	*d*
	*Motivational beliefs*						
1	Intrinsic goal orientation	5.74	0.73	5.79	0.72	NS	–
2	Task value	5.77	0.73	5.93	0.71	<0.001	0.22
3	Self‐efficacy	4.89	0.84	5.08	0.80	<0.001	0.23
	*Cognitive strategies*						
4	Elaboration	4.85	0.87	4.86	0.90	NS	–
5	Organisation	4.66	1.16	4.89	1.23	0.001	0.19
6	Metacognition	4.27	0.80	4.60	0.83	<0.001	0.40
	*Resource management*						
7	Time management	4.63	1.04	4.91	1.01	<0.001	0.27
8	Effort regulation	4.91	1.06	5.33	0.97	<0.001	0.41
	*Participation*					0.004	0.17
9	Lecture attendance	4.69	0.67	4.78	0.62	–	–
10	Study assignments	4.10	1.15	4.06	1.18	–	–
11	Skills training attendance	4.58	0.67	4.84	0.47	–	–
	*Year‐1 performance*						
12	Average grade	6.06	0.94	6.57	0.81	<0.001	0.57

*M* = mean; SD = standard deviation; NS = not significant.

**Table 2:** Cronbach's Alphas (on the diagonal in bold, for all cohorts combined) and Pearson correlations for the study variables (old cohorts [*n* = 648] above diagonal; new cohorts [*n* = 529] below diagonal)

	Variable	*n* Items	1	2	3	4	5	6	7	8	9	10	11	12
	*Motivational beliefs*													
1	Intrinsic goal orientation	4	***0.61***	0.61^a^	0.51^a^	0.45^a^	0.27^a^	0.39^a^	0.27^a^	0.32^a^	0.13^a^	0.10^b^	0.10^b^	0.11^a^
2	Task value	6	0.56^a^	***0.85***	0.43^a^	0.41^a^	0.33^a^	0.39^a^	0.33^a^	0.43^a^	0.20^a^	0.14^a^	0.14^a^	0.13^a^
3	Self‐efficacy	8	0.47^a^	0.39^a^	***0.87***	0.39^a^	0.17^a^	0.38^a^	0.36^a^	0.28^a^	0.06	0.05	0.03	0.18^a^
	*Cognitive strategies*													
4	Elaboration	6	0.44^a^	0.40^a^	0.33^a^	***0.68***	0.54^a^	0.60^a^	0.51^a^	0.42^a^	0.16^a^	0.22^a^	0.19^a^	0.17^a^
5	Organisation	4	0.20^a^	0.28^a^	0.08	0.53^a^	***0.74***	0.51^a^	0.42^a^	0.40^a^	0.21^a^	0.23^a^	0.13^a^	0.13^a^
6	Metacognition	10	0.34^a^	0.33^a^	0.29^a^	0.63^a^	0.48^a^	***0.77***	0.50^a^	0.46^a^	0.17^a^	0.21^a^	0.17^a^	0.17^a^
	*Resource management*													
7	Time management	5	0.24^a^	0.26^a^	0.30^a^	0.36^a^	0.34^a^	0.45^a^	***0.72***	0.69^a^	0.29^a^	0.52^a^	0.27^a^	0.32^a^
8	Effort regulation	4	0.31^a^	0.32^a^	0.20^a^	0.34^a^	0.29^a^	0.39^a^	0.60^a^	***0.74***	0.33^a^	0.51^a^	0.30^a^	0.35^a^
	*Participation*													
9	Lecture attendance	1	0.08	0.05	−0.04	0.08	0.07	0.04	0.17^a^	0.20^a^	–	0.33^a^	0.48^a^	0.33^a^
10	Study assignments	1	0.15^a^	0.14^a^	0.10^b^	0.18^a^	0.17^a^	0.19^a^	0.41^a^	0.40^a^	0.25^a^	–	0.30^a^	0.43^a^
11	Skills training attendance	1	0.15^a^	0.15^a^	0.07	0.05	0.03	0.06	0.13^a^	0.19^a^	0.45^a^	0.28^a^	–	0.29^a^
	*Year 1 performance*													
12	Average grade	–	0.01	0.02	0.09^b^	0.03	0.04	0.05	0.23^a^	0.25^a^	0.14^a^	0.38^a^	0.20^a^	–

a p < 0.01.

b p < 0.05.

### Differences in self‐regulated learning, participation and performance

The manova with assessment policy as independent variable (IV) and students’ scores on the eight separate subscales of the MSLQ, the three items for participation in scheduled learning activities and average grade as dependent variables (DVs), resulted in a highly significant Box's *M* (p < 0.001). Because Box's *M* test is sensitive to departures from normality, and the three participation variables were negatively skewed, we averaged the three participation variables into one participation variable and continued our analysis with this single participation variable. Thereafter, the assumptions for a manova were met. The multivariate test was significant for assessment policy (Pillai's Trace = 0.131, *F* [9, 1165] = 18.819, p < 0.001), indicating differences on the DVs between the assessment policies. Univariate analyses showed that students under the new assessment policy scored significantly higher on the measures task value (*F* [1, 1175] = 14.214, p < 0.001, *d* = 0.22), self‐efficacy (*F* [1, 1175] = 15.676, p < 0.001, *d* = 0.23), organisation (*F* [1, 1175] = 10.655, p = 0.001, *d* = 0.19), metacognitive self‐regulation (*F* [1, 1175] = 45.656, p < 0.001, *d* = 0.40), effort regulation (*F* [1, 1175] = 48.610, p < 0.001, *d* = 0.41), time management (*F* [1, 1175] = 21.154, p < 0.001, *d* = 0.27) and participation (*F* [1, 1175] = 8.554, p *=* 0.004, *d* = 0.17). Differences in average grade were also significant (*F* [1, 1175] = 99.554, p < 0.001, *d* = 0.57), with higher average grades for students under the new assessment policy. Hence, only differences in intrinsic goal orientation and elaboration were not statistically significant.

### Multi‐group analysis of structural relationships

Results from the multi‐group SEM indicated measurement invariance, because the CFI, RMSEA and SRMR were below the thresholds for proper model fit (see Table 3). Hence, the measurement models were equal between groups, indicating that the same factors were being measured under the old and new assessment policies. Additionally, the structural model (i.e. Model 3 versus Model 2) was not significantly different across groups, indicating that the structural relationships were similar in the old and new assessment policies. The final Model 3, with both measurement and structural invariance, had the smallest AIC (which is used to compare models) and showed good fit to the data (χ^2^[108, *n* = 1177] = 354.835, CFI = 0.947, SRMR = 0.048, RMSEA = 0.044), indicating that the model was invariant across assessment policies.

**Table 3:** Goodness‐of‐fit statistics for tests of measurement and structural invariance across old and new assessment policies

	Model description	Comparative model	χ^2^	df	CMIN/d.f.	Δdf	CFI	Δ CFI^a^	RMSEA	SRMR	AIC
1	Configural model; no equality constraints imposed	–	332.907	96	3.47	–	0.949	–	0.046	0.045	452.907
2	Measurement model; all factor loadings, error covariance and covariance constrained equally	2 versus 1	351.210	104	3.38	8	0.946	−0.003	0.045	0.048	455.210
3	Structural model; all factor loadings, error covariance, covariance and structural paths constrained equally	3 versus 2	354.835	108	3.29	4	0.947	0.001	0.044	0.048	450.835

CMIN/d.f. = chi‐squared divided by the degrees of freedom; CFI = comparative fit index; RMSEA = root mean square error of approximation; SRMR = standardised root mean squared residual; AIC = Akaike information criterion.

a ΔCFI should be less than 0.01.

**Figure 2:**
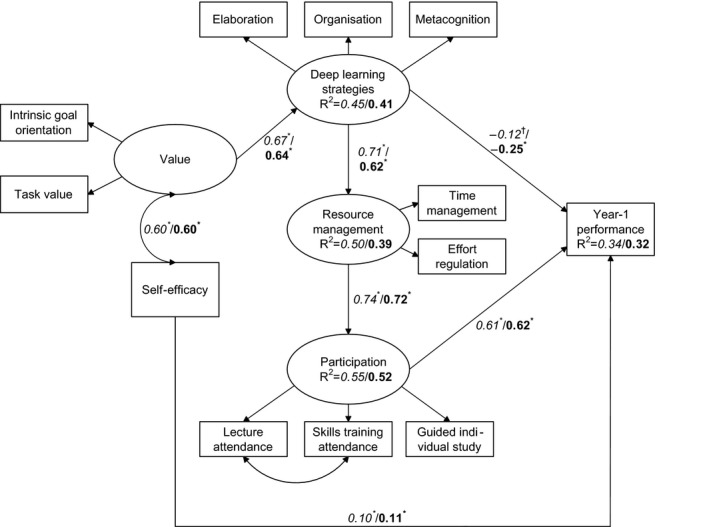
Multi‐group model of Year 1 performance. Observed variables are represented by rectangles, latent constructs are represented by ovals. Results are *italic* for old group and **bold** for new group. Reported path values are standardised regression weights. *p < 0.001 and ^†^p < 0.05, indicate whether the structural relationship per group is significant. *R* ^2^ is the proportion of variance accounted for that specific variable.

Consequently, there was a positive path from value through deep learning, resource management and participation to Year‐1 performance (see Fig. 2). There also was a negative direct relationship between deep learning and average grade, whereas self‐efficacy showed a positive direct relation with average grade. The model explained 34% of the variance in average grades for students under the old assessment policy, and 32% of the variance for students under the new assessment policy.

## Discussion

This study showed that average grades were superior under a new assessment policy with higher stakes and higher performance standards, compared with an old policy with lower stakes and lower performance standards. Task value, self‐efficacy, organisation, metacognition, effort regulation, time management and participation were significantly higher under the new policy, but intrinsic goal orientation and elaboration did not differ between the assessment policies. Additionally, the effect sizes for metacognition (*d* = 0.40), effort regulation (*d* = 0.41) and academic performance (*d* = 0.57) were substantial. The structural relationships between SRL, participation and academic performance were invariant, indicating that the relationships between SRL, participation and academic performance are similar in the two assessment policies. Thus, it seems that the higher academic performance under the new assessment policy can be explained by increases in SRL and participation compared with the old assessment policy, although the ways in which SRL and participation affect performance are similar in both policies.

### Higher academic performance, self‐regulated learning and participation

It is not surprising that academic performance improved after the stakes and performance standards were raised, because this is in line with previous findings.^4^, ^5^, ^6^, ^7^, ^8^ However, the magnitude of the increase in performance is striking, because it is identical to the rise in performance standards (i.e. half a point on a 10‐point scale). This suggests that students are highly responsive to the minimal performance standards. It would therefore be interesting to further investigate the relation between demands of the assessment policy and academic performance.

Perhaps more surprising than the rise in performance, were the higher average scores for the new cohorts on the motivational construct task value, and the lack of a difference in intrinsic goal orientation. These results seem to contradict the notion that extrinsic motivators decrease, or have no influence on, intrinsic motivation.^38^, ^39^ A possible explanation is that the number of extrinsic motivators, in this case examinations, has not been raised. Only the characteristics (i.e. the stakes and performance standards) of the extrinsic motivators were altered, and perhaps these characteristics now better match the students’ performance level, as indicated by the higher self‐efficacy of students under the new assessment policy. In other words, specific difficult goals can be motivating, as long as the goals are deemed important and attainable.^40^

Concerning self‐regulated learning strategies and participation, we found higher scores on measures of deep learning (i.e. organisation and metacognitive self‐regulation), resource management (i.e. time and study environment, and effort regulation) and participation for the new cohorts. An explanation that needs further examination is that when stakes and performance standards are raised, students increase the frequency of learning behaviours by which they expect to achieve success. The fact that elaboration did not increase significantly would then indicate that students judge elaboration to be less important for achieving high grades. Overall, we found higher academic performance, SRL and participation under the new assessment policy, compared with the old policy.

### Similar relationships in both assessment policies

The structural relationships between SRL, participation and academic performance in the model were comparable across both assessment policies, which indicates that SRL and participation were similarly related to academic performance under both policies. In short, higher value is associated with higher deep learning, which is related to better resource management, higher participation and better academic performance. Self‐efficacy shows a positive direct relation with academic performance. However, there is also a negative direct link from deep learning to academic performance. This may indicate that the Year‐1 assessments do not reward deep learning, or alternatively that students need to combine deep learning with proper resource management and participation, in order to achieve academic success.^13^ Our results are somewhat surprising, because earlier research reported that when the stakes are raised, motivation shows weaker relationships with academic performance, and learning strategies and metacognitive strategies are more strongly related to academic performance.^4^, ^22^ However, we compared high stakes with even higher stakes, whereas these earlier studies compared low stakes with high stakes. In sum, it seems that SRL and participation are associated with academic performance in the same way under both assessment policies.

### Limitations

The current study has several limitations that need to be addressed. First, we used correlational data, hence no firm causal conclusions can be drawn. Second, we used student responses on self‐report questionnaires as measures of learning behaviours, which might be influenced by social desirability. Nonetheless, responding to the questionnaire was voluntary and confidential and the primary goal of the questionnaire was to aid students in self‐reflection on the strengths and weaknesses of their study approach. Therefore, we do not expect answers to be shaped by social desirability. Third, we should note that the percentage of early dropouts was higher under the new assessment policy (19%), compared with the old policy (7%). As it is likely that mainly students with low scores on our study variables dropped out, this might partly explain the average differences between students under both assessment policies. However, we were able to check for differences on the basis of early dropouts who did complete the questionnaire, and still found comparable differences between the assessment policies on the study variables when they were included. Also, the standard deviations of our study variables were highly similar across both assessment policies, which contradicts the notion that only students with low scores on these variables dropped out. Moreover, this early selection is an effect the assessment policy may have, discouraging some students from continuing their study while improving grades for those who stay.^41^

Another limitation of this study is the fact that we could only compare the results for the 2014 and 2015 cohorts with those for the cohorts from 2008 and 2009, because the MSLQ and participation questionnaire was not conducted in the years 2010 to 2013. Although no major alterations in the curriculum were made in this period, the selection procedure was changed in 2012. For the 2008/2009 cohorts 50% of students were admitted by weighted lottery and 50% were selected by a school‐specific selection procedure; for an explanation of this procedure see Stegers‐Jager et al.^42^ For the cohorts since 2012 these numbers were 20% and 80%, respectively. However, we do not expect this time gap or altered selection procedure to have influenced the results. First, research shows no differences in pre‐university grade point average and Year‐1 achievement between selected and lottery‐admitted students.^43^ Second, we were able to compare the average Year‐1 grades for the 2012 and 2013 cohorts (i.e. the last cohorts under the old assessment policy) with those for the 2014 and 2015 cohorts, and found differences similar to those reported in the current study: the average grades for the 2012 and 2013 cohorts (*M* = 6.09, SD = 0.97) did not differ significantly from the average grades for the 2008 and 2009 cohorts (*M* = 6.06, SD = 0.94), but were significantly different from those for the 2014 and 2015 cohorts (*M* = 6.57, SD = 0.81) (*t* (750) = −13.691, p < 0.001). In sum, the significant change in academic performance did not seem to coincide with the change in selection procedure, but with the change in assessment policy.

### Practical implications and suggestions for further research

An important practical implication of this study is that medical schools should be keenly aware of the influence their assessment policy has on student learning and academic performance. Although intrinsic motivation is important, external triggers may have a powerful additional effect on academic motivation.^44^ Developing an assessment policy that boosts motivation might be an efficient way to challenge students to perform better. A meta‐analysis showed that the goals that students have in terms of grades are one of the most important predictors of academic performance.^16^ Although it seems likely that the stakes and performance standards will influence these grade goals, the connection of the assessment policy to students’ grade goals and subsequent academic performance needs further exploration. Additionally, it would be interesting to separate the effects of higher stakes and the effects of higher performance standards on academic performance, in order to compare their relative contribution. Finally, in order to fully understand the effects of higher stakes and performance standards, an investigation of the long‐term consequences of these alterations is necessary. Many tests do not capture the full range of competencies and knowledge,^45^ or may negatively affect the motivation to learn, especially when the tests are high stakes.^39^ Therefore, although we found higher task value and no differences in intrinsic goal orientation under the new assessment policy, it is important to monitor motivation for learning in the long term as well.

## Conclusion

In conclusion, overall we found higher academic performance, SRL and participation for students under the new assessment policy compared with the old policy with lower stakes and lower performance standards, but no differences in intrinsic goal orientation and elaboration. Structural relationships between SRL, participation and performance were not different between the assessment policies, indicating that the relation of academic performance to these constructs is similar in both assessment policies. Thus, although SRL, participation and performance are higher under the new assessment policy, their associations remain the same. Hence, these results underscore the literature, showing that SRL and participation are important for explaining academic performance. In addition, it seems that this relation is relatively stable under different assessment policies and, most importantly, that SRL, participation and performance can be improved by the design of assessment policies. In sum, characteristics of the assessment policy seem to play an important role in optimising student learning and academic performance.

## Contributors

All authors contributed substantially to the conception and design of the study. RK and KMS‐J collected the data; RK analysed the data. All authors contributed to the interpretation of the data. RK wrote the first draft and all authors critically revised it for important intellectual content. All authors approved the final version of the manuscript to be published. All authors are accountable for all aspects of the work and ensuring that questions related to the accuracy or integrity of any part of the study are appropriately investigated and resolved.

## Funding

None.

## Conflicts of interest

None.

## Ethical approval

The ethical committee of the Department of Psychology, Education and Child Studies of Erasmus University Rotterdam deemed further approval of a Medical Ethical Evaluation committee to be not required.
